# Placental Metabolism Is Linked to Prenatal Vitamin Supplement Use in the First Month of Pregnancy in the MARBLES Cohort

**DOI:** 10.1016/j.tjnut.2025.05.016

**Published:** 2025-05-23

**Authors:** Mariana Parenti, Rebecca J Schmidt, Daniel J Tancredi, Meghan Miller, Irva Hertz-Picciotto, Cheryl K Walker, Carolyn M Slupsky

**Affiliations:** 1 Department of Nutrition, University of California, Davis, CA, United States; 2 Department of Public Health Sciences, University of California, Davis, CA, United States; 3 MIND Institute, University of California, Davis, CA, United States; 4 Department of Pediatrics, School of Medicine, University of California, Davis, CA, United States; 5 Department of Psychiatry & Behavioral Sciences, School of Medicine, University of California, Davis, CA, United States; 6 Department of Obstetrics & Gynecology, School of Medicine, University of California, Davis, CA, United States; 7 Department of Food Science and Technology, University of California, Davis, CA, United States

**Keywords:** prenatal supplementation, metabolomics, placenta, umbilical cord serum, neurodevelopment

## Abstract

**Background:**

The first month of pregnancy is a key time in early developmental programming. Prenatal vitamin/mineral supplement use during the first month of pregnancy (PNVmo1) was associated with reduced risk of autism spectrum disorder (ASD) in the Markers of Autism Risk in Babies, Learning Early Signs (MARBLES) cohort.

**Objectives:**

We aimed to evaluate the associations between PNVmo1, the placental and umbilical cord serum metabolomes, and the child’s later neurodevelopmental outcome in the MARBLES pregnancy cohort.

**Methods:**

Placental (*n* = 78) and umbilical cord serum (*n* = 132) metabolomes were investigated using ^1^H nuclear magnetic resonance spectroscopy. PNVmo1 was determined by self-report. At 36 mo of age, child neurodevelopmental outcomes were classified by MARBLES clinicians into 3 groups: typically developing (TD), ASD, or nontypically developing (Non-TD) but not ASD, which was dominated by developmental delays and/or elevated autism symptoms but not meeting ASD criteria.

**Results:**

After adjustment for covariates, permutational multivariate analysis of variance revealed that PNVmo1 was significantly (*P* < 0.05) associated with the placental and umbilical cord serum metabolomes. In the placenta, higher concentrations of amino acids were observed in the PNVmo1 group (false discovery rate <0.1). After adjustment for PNVmo1 and other covariates, permutational multivariate analysis of variance revealed a significant association (*P* < 0.05) between the placental metabolome and Non-TD outcome status. No associations were observed in the analyses of umbilical cord serum metabolism or with ASD outcome. We tested for but did not find evidence that the placental metabolome explained the relationship between PNVmo1 and Non-TD outcome in an exploratory mediation analysis.

**Conclusions:**

These findings suggest that the placental metabolome could be sensitive to nutrient supplementation during the earliest stages of pregnancy.

## Introduction

Nutrition before and during pregnancy has long-term health effects on the child, including impacts on neurodevelopment [1]. For instance, maternal folate status has been associated with the development of the fetal brain and national folic acid fortification programs have been undertaken to reduce the incidence of neural tube defects [2]. In the crucial window around conception, maternal nutrition status has been recognized as a factor that might modify autism spectrum disorder (ASD) likelihood [3,4]. Prenatal vitamin/mineral supplement (PNV) use during the first month of pregnancy (PNVmo1) has been associated with reduced likelihood of ASD familial recurrence in younger siblings of children with ASD in the Marker of Autism Risk in Babies, Learning Early Signs (MARBLES) cohort [5]. It is plausible that PNVmo1 influences neurodevelopment through improved nutritional status, as PNV use is associated with reduced risk of prenatal dietary inadequacy for vitamins and minerals compared with micronutrient intake from food alone [6]. Analysis of dietary intake from food alone among pregnant women in the United States revealed a risk of dietary inadequacy for a number of micronutrients [6]. The risk of dietary inadequacy from food alone differs by maternal age, race/ethnicity, and education [7]. Thus, PNVmo1 might improve maternal nutritional status during a crucial developmental window.

The placenta plays a central role in transferring nutrients from mother to fetus and it is highly sensitive to maternal nutritional status [4,8,9]. Both maternal folic acid and PNV use in early pregnancy have been linked to placental function, including lower uteroplacental vascular resistance, as well as placental epigenetic changes, such as global hypomethylation and key methylation sites related to neuronal developmental pathways [[10], [11], [12], [13], [14]]. Insults, including over- and undernutrition, could disrupt the sequence of placental growth and development over the course of pregnancy, ultimately disrupting fetal development [1]. Indeed, placental development has been linked to brain development through a placenta–brain axis [15]. We have also recently shown that placental and umbilical cord serum metabolic profiles are highly correlated in the MARBLES cohort [16]. Thus, our aim was to investigate the associations between PNVmo1, neurodevelopmental outcomes, and the placental and umbilical cord serum metabolomes using proton nuclear magnetic resonance (^1^H NMR) spectroscopy-based metabolomics in a subset of the MARBLES cohort.

## Methods

### Study population

This analysis included participants from the ongoing MARBLES prospective cohort which began enrollment in 2006 [17]. Women with ≥1 child diagnosed with ASD were enrolled prior to or early during pregnancy, followed through pregnancy and their child's early life, and their child's cognitive and behavioral development was subsequently assessed. The University of California, Davis, institutional review board and the California Committee for the Protection of Human Subjects approved this study and the MARBLES study protocols. All participants provided written informed consent prior to collection of data and specimens. Information related to PNV use during the 6 mo prior to pregnancy and in each month of pregnancy was collected by telephone interview as previously described [5]. In MARBLES, PNV use was common, with 95.9% of participants reporting using PNV at some point during pregnancy [5]. In this analysis, the exposure PNVmo1 was defined as reported prenatal vitamin/mineral supplement use during the first month of pregnancy because PNV use in this period was associated with reduced likelihood of ASD recurrence in younger siblings in MARBLES [5].

In a previous analysis, we identified PNVmo1 as a potential confounder of the relationships between an environmental exposure, the placental metabolome, and neurodevelopmental outcomes [18]. However, to our knowledge, a relationship between periconceptional or early pregnancy PNV use and the placenta or umbilical cord blood metabolomes had not been reported. Thus, to investigate an association between PNVmo1 and the placenta metabolome, we identified placenta samples *1*) excluded from the prior analysis because they lacked data related to the environmental exposure and *2*) which had complete covariate data (*N* = 78).

Given our previous report showing a significant association between the placental and umbilical cord serum metabolomes [16], we hypothesized that changes observed in the placental metabolome might also be reflected in fetal blood. Thus, we also investigated the association between PNVmo1 and the umbilical cord serum metabolome. A total of 16 samples were excluded: 2 were due to missing PNV use data, 3 due to missing covariate data, 4 due to missing neurodevelopmental outcome data, and a further 7 due to having an older sibling in the analysis. In total, this analysis included *N* = 132 umbilical cord serum samples.

### Assessment of child neurodevelopmental outcomes

At ∼3 y of age, children were classified into 3 neurodevelopmental outcome groups as previously described, which included typically developing (TD), ASD, and nontypically developing (Non-TD) [19]. MARBLES clinicians assessed the children for ASD using the gold-standard ASD Diagnostic Observation Schedule (ADOS) [20]. Additionally, the Mullen Scales of Early Learning (MSEL) were used to assess verbal and nonverbal development [21]. Scores on both the ADOS and MSEL at ∼36 mo were used to classify neurodevelopmental outcomes. Children who received a clinical best estimate outcome classification of ASD based on *Diagnostic and Statistical Manual of Mental Disorders, Fifth edition* criteria and who had an ADOS score ≥ASD cutoff were classified as ASD. Children without ASD but who had ADOS scores within 3 points of the ASD cutoff and/or had ≥1 MSEL subdomain score 2 SDs below the mean and/or had ≥2 MSEL scores 1.5 SDs below the mean were classified as Non-TD. Other children not classified as ASD or Non-TD in the study were classified as TD.

### Placental and umbilical cord serum sample preparation

Full-thickness placental tissue sections were collected at delivery and stored at –80°C. All samples were partially thawed, and a 6 mm biopsy punch was taken as an aliquot for metabolic analysis and subsequently stored at –80°C until analysis. Placental samples were extracted as previously described [16,18]. Briefly, placenta samples were ground under cryogenic conditions using liquid nitrogen and extracted using a modified Folch extraction [22]. The polar metabolite layer was collected, dried, and reconstituted in 10 mM potassium phosphate buffer. An internal standard (Chenomx), containing 5.0 mM 3-(trimethylsilyl)-1-propanesulfonic acid-d6 (DSS-d6), 0.2% NaN_3_, and 99.8% D_2_O, was added to enable locking and absolute quantification. Each sample’s pH was adjusted to 6.8 ± 0.1, 180 μL was loaded into a 3-mm NMR tube (Bruker), and samples were stored at 4 °C until spectral acquisition the same day.

Umbilical cord blood was collected at delivery, centrifuged, and the resulting serum was collected and stored at –80°C until preparation for metabolomics analysis. Umbilical cord serum samples were prepared for metabolomics analysis as previously described [16]. Samples were thawed on ice, underwent ultrafiltration centrifugation to remove protein using Amicon Ultra-0.5 mL 3000 MW centrifugal filters (Millipore), and the resulting filtrate was measured and frozen. Frozen filtrate was dried under vacuum using a miVac concentrator system (Genevac), and reconstituted using 240 μL of 10 mM potassium phosphate buffer prepared in D_2_O to improve signal to noise. The pH of the buffer was adjusted to 6.8 ± 0.1 before sample reconstitution. Finally, 180 μL of each sample was loaded into 3-mm NMR tubes (Bruker) and stored at 4°C until spectral acquisition the same day.

### ^1^H NMR metabolomics analysis

^1^H-NMR spectra were captured using the noesypr1d pulse sequence with a Bruker Avance 600 MHz spectrometer (Bruker) as previously described [18]. We manually phase- and baseline-corrected spectra using Chenomx NMR Processor (version 8.1, Chenomx) and quantified metabolite concentrations using Chenomx Profiler (version 8.1). The quantification relies on the known concentration of the internal standard DSS-d6 to determine the concentration of each metabolite using a library of compound spectral signatures [23,24]. This enabled us to identify and quantify compounds within an NMR spectrum.

### Statistical analysis

All statistical analyses were conducted using the R statistical language (v. 4.4.0) with RStudio (v. 2024.04.1). Demographic differences between subjects were assessed using χ^2^ tests for categorical variables and Mann–Whitney *U* tests for continuous variables. We identified and quantified 62 placental metabolites and 50 umbilical cord serum metabolites. Metabolites that might have been introduced during sample preparation (such as glycerol for umbilical cord serum or methanol for placental tissue) or that were detected in <80% of samples were excluded from statistical analysis, as described elsewhere [16,18]. Metabolite concentrations were corrected for dilution and are reported as nmol/g placental tissue or μmol/L for cord serum. Concentrations of 54 placental and 44 cord serum metabolites were log_10_(x+1)-transformed to improve skewed distributions.

Our primary interest was in testing the multivariate association between PNVmo1 and each metabolome, allowing investigation of complex relationships within high-dimensional metabolomics data. The association of PNVmo1 with each metabolome (umbilical cord and placental) was assessed using permutational multivariate analysis of variance (PERMANOVA) adjusted for potential confounders [25]. In each case, we first tested for and found no differences in dispersion, which can confound the results of PERMANOVA [25]. Much like multivariate analysis of variance, PERMANOVA is used to test for differences in group centroids but is robust to non-normal data. When the metabolome-wide association of PNVmo1 was significant (*P* < 0.05), we assessed its association with individual metabolites with single-response PERMANOVA to provide insight into the metabolites contributing to the multivariate results. For individual metabolites, these results were corrected for false discovery rate (FDR) using the Benjamini–Hochberg procedure [26]. We set our threshold at FDR < 0.1 in alignment with previous analyses [16,18]. The metabolome-wide and individual metabolite associations between neurodevelopmental outcome and each metabolome were similarly assessed with PERMANOVA and single-response PERMANOVA, adjusted for covariates, and FDR-corrected. PERMANOVA was implemented using the package *vegan* [27]. To identify potential confounders, we used a directed acyclic graph (DAG, Supplemental Figure 1) to encode our assumptions and identify covariates a priori using *dagitty* [28]. The DAG included birth year, fetal sex, maternal age (years), maternal educational attainment (bachelor's degree or more, or some college or less), maternal self-reported race and ethnicity as a proxy for experiencing systemic inequity (non-Hispanic White or historically marginalized groups), gestational age at delivery (wk), maternal metabolic conditions (including prepregnancy obesity, gestational diabetes, hypertension, and preeclampsia), and home ownership as a proxy of socioeconomic status. Final models to evaluate the association between PNVmo1 and the metabolomes were adjusted for birth year, fetal sex, maternal age, maternal education, maternal self-reported race and ethnicity, home ownership, and maternal metabolic conditions. We then developed additional models to evaluate the associations between neurodevelopmental outcomes and the metabolomes that were adjusted for those same variables and PNVmo1. Given the role of gestational age at birth as a collider in perinatal research and a potential mediator that can introduce substantial bias [29] when addressing total effects regardless of gestational age, it was not included in this model.

To test for potential mediation of the association between PNVmo1 and neurodevelopmental outcome group by the metabolomes, we used redundancy analysis (RDA, function *vegan::rda*) as a supervised dimension reduction technique to extract a latent variable that maximizes the variation in the metabolome associated with PNVmo1 [30]. The RDA latent variable can be used to project metabolite loadings, which we used to identify important metabolites associated with PNVmo1, and participant scores, which we used to model the placental metabolome in mediation analysis. Mediation analyses were conducted using the package *mediation* [31]. The models included the covariates birth year, fetal sex, maternal age, maternal education, maternal self-reported race and ethnicity, home ownership, and maternal metabolic conditions. To reduce the number of small or empty cells, in this analysis, maternal metabolic conditions were collapsed into a dichotomous variable using the group with no metabolic conditions as the reference. Because the sum of squares of the metabolite loadings for the RDA latent variable is equal to 1, metabolites whose squared loading was >$\frac{1}{54}$ described more variation than average and thus were considered important. Placental metabolites with FDR < 0.1 and whose squared loading was >$\frac{1}{54}$ were identified as important contributors to the multivariate association between the placental metabolome and PNVmo1. Graphics were generated with the package *ggplot2* [32].

## Results

Characteristics of mother–child dyads by PNVmo1 are presented in Table 1. In general, dyads with PNVmo1 exposure reported higher maternal educational attainment than dyads without PNVmo1 exposure. The analysis of the placental metabolome included 36 dyads with PNVmo1 exposure and 42 participants without PNVmo1. Participants with PNVmo1 exposure were more likely to self-identify as non-Hispanic White. The TD, ASD, and Non-TD groups had 46, 22, and 10 dyads, respectively. The analysis of the umbilical cord serum metabolome included 65 dyads with PNVmo1 exposure and 67 dyads with no PNVmo1 exposure. In this analysis, neurodevelopmental outcome was associated with PNVmo1 exposure, and the TD, ASD, and Non-TD groups had 72, 43, and 17 dyads, respectively. In this sample, there was no difference in maternal self-reported race and ethnicity by PNVmo1 exposure. However, home ownership was more common with PNVmo1 exposure.

**TABLE 1:** Participant characteristics by prenatal vitamin use in the first month of pregnancy (PNVmo1) for each subanalysis.

Characteristics	Placenta analysis			Umbilical cord serum analysis		
	No PNVmo1 (*N* = 42)	Yes PNVmo1 (*N* = 36)	*P* value	No PNVmo1 (*N* = 67)	Yes PNVmo1 (*N* = 65)	*P* value
Maternal age at birth (y)			0.07			0.80
Median	35.9	32.8		34.5	34.2	
IQR	32.2–38.5	30.8–37.1		30.7–37.9	31.1–38.0	
Prepregnancy BMI			0.41			0.22
Median	26.6	27.8		25.095	25.843	
IQR	22.9–30.6	23.3–32.6		22.4–29.1	23.1–31.0	
Birth year			0.28			0.65
Median	2015	2015		2013	2013	
IQR	2014–2016	2014–2015		2010–2014	2011–2014	
Gestational age at birth (wk)			0.31			0.33
Median	39.3	39.1		39.1	39.0	
IQR	39.0–39.7	38.6–39.9		38.8–39.9	38.4–39.7	
Maternal education, *n* (%)			0.04			<0.001
Bachelor’s degree or higher	16 (38.1)	22 (61.1)		23 (34.3)	42 (64.6)	
Some college or less	26 (61.9)	14 (38.9)		44 (65.7)	23 (35.4)	
Maternal self-reported race, *n* (%)			0.04			0.48
Non-Hispanic White	16 (38.1)	22 (61.1)		33 (49.3)	36 (55.4)	
Historically marginalized groups^1^	26 (61.9)	14 (38.9)		34 (50.7)	29 (44.6)	
Home ownership, *n* (%)			0.17			0.002
No	24 (57.1)	15 (41.7)		39 (58.2)	20 (30.8)	
Yes	18 (42.9)	21 (58.3)		28 (41.8)	45 (69.2)	
Maternal metabolic conditions, *n* (%)			0.95			0.39
BMI < 25, no metabolic conditions	12 (28.6)	11 (30.6)		27 (40.3)	18 (27.7)	
25 ≤ BMI < 30, no metabolic conditions	11 (26.2)	7 (19.4)		17 (25.4)	18 (27.7)	
BMI ≥ 30, no metabolic conditions	6 (14.3)	7 (19.4)		8 (11.9)	14 (21.5)	
Any hypertensive disorder without diabetes, at any BMI	5 (11.9)	4 (11.1)		5 (7.5)	3 (4.6)	
Any diabetes, at any BMI	8 (19.0)	7 (19.4)		10 (14.9)	12 (18.5)	
Child’s sex, *n* (%)			0.25			0.62
Male	23 (54.8)	15 (41.7)		42 (62.7)	38 (58.5)	
Female	19 (45.2)	21 (58.3)		25 (37.3)	27 (41.5)	
Neurodevelopmental Outcome, *n* (%)			0.43			0.03
TD	22 (52.4)	24 (66.7)		29 (43.3)	43 (66.2)	
ASD	14 (33.3)	8 (22.2)		28 (41.8)	15 (23.1)	
Non-TD	6 (14.3)	4 (11.1)		10 (14.9)	7 (10.8)	

Continuous variables are presented as the median and IQR, and categorical variables are presented as count (%). Significance was tested using either the Mann–Whitney *U* test, or the chi-squared test (for categorical variables).

Abbreviations: ASD, autism spectrum disorder; Non-TD, nontypically developing; TD, typically developing.

1 Maternal self-reported race and ethnicity was originally reported as Asian, Black or African American, Hispanic non-White, Hispanic White, Pacific Islander, multiracial, or White. The variable was collapsed to reduce small cells and was used as a proxy for structural inequity in subsequent analyses.

### Associations between PNVmo1 and metabolomics

We first investigated the multivariate association between PNVmo1 and the placental metabolome using PERMANOVA. We tested for and did not find differences in beta-dispersion by PNVmo1 (*P* = 0.87). In models adjusted for birth year, fetal sex, maternal age, maternal education, maternal self-reported race and ethnicity, home ownership, and maternal metabolic conditions, PNVmo1 explained a small but significant portion of the variation in the placental metabolome (*R*^*2*^ = 0.034, *P* = 0.0085 under 9999 permutations). We then investigated the associations between individual placental metabolites and PNVmo1 (Table 2). We observed differences (FDR < 0.1) for 22 metabolites, including amino acids and their derivatives.

**TABLE 2:** Associations between prenatal vitamin use in the PNVmo1 and placental metabolite concentrations (nmol/g).

Metabolite	No PNVmo1^1^ (*N* = 42)	PNVmo1^1^ (*N* = 36)	*R*^*2*^^2^	*P* value	FDR
1,3-Dihydroxyacetone	16.4 (7.7, 30.7)	15.3 (8.0, 33.5)	0.0011	0.7739	0.8037
2-Hydroxybutyrate	25.8 (18.2, 33.7)	32.3 (27.1, 36.1)	0.0392	0.0601	0.1269
3-Hydroxybutyrate	150.9 (69.9, 201.3)	197.6 (97.4, 384.0)	0.0284	0.1452	0.2306
4-Aminobutyrate	22.5 (16.9, 32.0)	28.6 (19.7, 35.0)	0.0481	0.0650	0.1300
4-Hydroxybutyrate	37.3 (20.5, 45.8)	40.6 (25.3, 61.5)	0.0212	0.1989	0.2692
Acetate	262.8 (190.6, 340.1)	279.6 (228.2, 328.5)	0.0186	0.1956	0.2692
Alanine	853.8 (787.6, 976.5)	977.0 (820.3, 1319.4)	0.1197	0.0011	0.0248^3^
Arginine	180.9 (149.4, 212.6)	177.6 (148.1, 245.5)	0.0244	0.1526	0.2354
Asparagine	156.3 (125.9, 185.7)	172 (143.0, 204.7)	0.0960	0.0055	0.0248^3^
Aspartate	1004.4 (813.5, 1183.9)	973.8 (846.6, 1200.0)	0.0017	0.7254	0.7737
Betaine	28.7 (20.4, 34.5)	31.2 (25.2, 35.8)	0.0471	0.0541	0.1217
Carnitine	127.8 (99.6, 154.0)	141.4 (113.5, 173.9)	0.0474	0.0611	0.1269
Choline	1630.4 (1307.5, 1830.1)	1639.8 (1497.7, 2086.5)	0.0404	0.0783	0.1458
Creatine	385.0 (309.5, 484.5)	429.1 (375.3, 524.3)	0.0293	0.1432	0.2306
Cystine	36.3 (25.0, 46.3)	35.3 (26.6, 50.1)	0.0546	0.0399	0.0979^3^
Ethanolamine	976.0 (763.0, 1125.5)	1022.5 (884.8, 1234.4)	0.0369	0.0836	0.1505
Formate	191.6 (171.0, 237.6)	202.6 (171.8, 217.5)	0.0120	0.3299	0.4049
Fumarate	36.1 (26.0, 53.4)	35.9 (29.3, 52.2)	0.0003	0.8894	0.8894
Glucitol	930.4 (685.4, 1220.1)	928.9 (768.2, 1166.5)	0.0065	0.4782	0.5614
Glutamate	2567.6 (2241.5, 2875.7)	2759.3 (2470.6, 3138.9)	0.0673	0.0244	0.0669^3^
Glutamine	868.0 (743.8, 938.8)	917.8 (846.4, 1017.6)	0.0469	0.0515	0.1209
Glutathione	107.7 (84.8, 141.2)	94.5 (51.8, 141.1)	0.0364	0.0768	0.1458
Glycerol	1759.4 (1385.8, 2138.1)	2040.6 (1628.1, 2427.4)	0.0672	0.0257	0.0669^3^
Glycine	1069.8 (911.6, 1333.7)	1223.7 (1049.9, 1421.0)	0.1103	0.0028	0.0248^3^
Hypoxanthine	585.0 (475.6, 730.6)	660.4 (548.7, 767.0)	0.0628	0.0260	0.0669^3^
Inosine	223.3 (149.6, 322.6)	239.5 (172.8, 296.5)	0.0295	0.1154	0.2010
Isoleucine	168.6 (142.6, 184.9)	171.0 (144.2, 191.9)	0.0936	0.0041	0.0248^3^
Kynurenine	23.8 (15.4, 39.3)	32.6 (19.7, 44.5)	0.0270	0.1654	0.2481
Lactate	18,432.6 (16,562.0, 20,636.4)	20,806.2 (17,983.4, 23,658.7)	0.0899	0.0080	0.0328^3^
Leucine	279.9 (249.5, 335.0)	302.6 (260.1, 353.1)	0.1027	0.0035	0.0248^3^
Lysine	295.3 (221.0, 330.1)	305.7 (255.7, 341.9)	0.1032	0.0022	0.0248^3^
Methionine	84.2 (75.6, 100.8)	90.8 (74.6, 109.2)	0.0951	0.0054	0.0248^3^
*myo*-Inositol	1439.9 (1297.3, 1754.2)	1602.9 (1297.9, 1747)	0.0026	0.6497	0.7309
*N*-Acetylneuraminate	258.0 (184.1, 317.0)	276.9 (223.8, 345.0)	0.0046	0.5669	0.6513
NAD^+^	59.8 (47.2, 83.6)	60.7 (47.9, 80.3)	0.0132	0.2176	0.2798
Niacinamide	71.9 (48.0, 88.9)	74.5 (58.3, 98.0)	0.0228	0.2084	0.2745
*O*-Acetylcarnitine	66.6 (58.7, 91.9)	75.8 (63.9, 99.8)	0.0017	0.7239	0.7737
*O*-Phosphocholine	133.7 (107.8, 176.8)	128.1 (82.6, 177.2)	0.0848	0.0049	0.0248^3^
*O*-Phosphoethanolamine	484.6 (347.3, 606.7)	540.2 (232.4, 695.5)	0.0193	0.1994	0.2692
Ornithine	50.8 (40.1, 63.7)	51.7 (44.3, 59.7)	0.0718	0.0183	0.0581^3^
Pantothenate	17.1 (13.0, 24.4)	16.5 (13.2, 22.8)	0.0005	0.8437	0.8596
Phenylalanine	161.1 (145.0, 186.0)	183.1 (150.7, 196.7)	0.1116	0.0014	0.0248^3^
Proline	359.7 (313.0, 409.7)	368.1 (319.5, 439.6)	0.0833	0.0106	0.0382^3^
Pyroglutamate	45.4 (35.8, 62.5)	51.2 (37.6, 67.1)	0.0698	0.0144	0.0486^3^
Serine	439.6 (368.2, 490.7)	454.2 (368.1, 563.5)	0.0765	0.0085	0.0328^3^
*sn*-Glycero-3-phosphocholine	151.4 (98.6, 204.2)	161.3 (88.7, 331.1)	0.0160	0.2300	0.2888
Succinate	214.0 (147.2, 268.2)	232.6 (187.0, 277.7)	0.0061	0.4754	0.5614
Taurine	3047.3 (2358.1, 3564.0)	3235.2 (2778.6, 3706.1)	0.0210	0.1887	0.2692
Threonine	415.2 (352.0, 487.0)	465.2 (409.4, 558.4)	0.1000	0.0052	0.0248^3^
Tryptophan	22.8 (13.3, 28.1)	22.1 (15.1, 30.7)	0.0278	0.1302	0.2197
Tyrosine	191.3 (166.1, 213.4)	200.0 (167.2, 228.7)	0.1009	0.0032	0.0248^3^
Uracil	299.4 (241.2, 423.0)	353.4 (257.9, 503.5)	0.0686	0.0251	0.0669^3^
Uridine	118.2 (83.1, 148.3)	127.4 (100.9, 157.2)	0.0023	0.7307	0.7737
Valine	382.7 (330.0, 429.5)	407.7 (349.2, 441.3)	0.1168	0.0019	0.0248^3^

Abbreviations: PNVmo1, prenatal vitamin or mineral supplement use in the first month of pregnancy; No PNVmo1, no prenatal vitamin or mineral supplement use in the first month of pregnancy; FDR, false discovery rate; *R*^*2*^, partial coefficient of determination.

1 Median and IQR.

2 PERMANOVA was conducted on log-transformed metabolite concentrations under 9999 permutations. All models were adjusted for birth year, maternal age, maternal self-reported race and ethnicity, home ownership, maternal education, maternal metabolic condition, and fetal sex.

3 FDR < 0.1.

We also investigated the multivariate association between PNVmo1 and the umbilical cord serum metabolome using PERMANOVA. We tested for and did not find differences in beta-dispersion by PNVmo1 (*P* = 0.20). In the covariate-adjusted model, PNVmo1 was significantly associated with the cord serum metabolome (*R*^*2*^ = 0.014, *P* = 0.0451 under 9999 permutations). We then investigated the associations between individual placental metabolites and neurodevelopmental outcomes (Supplemental Table 1). However, no individual metabolites were associated with neurodevelopmental outcome at the FDR < 0.1 threshold.

### Associations between metabolomics and neurodevelopmental outcome

We then investigated the multivariate association between the placental metabolome and neurodevelopmental outcomes using PERMANOVA. We tested for and did not find differences in beta-dispersion by neurodevelopmental outcome group (*P* = 0.25). In the covariate-adjusted model, neurodevelopmental outcome group explained a small but significant portion of the variation in the placental metabolome (*R*^*2*^ = 0.048, *P* = 0.0169 under 9999 permutations). Pairwise comparisons revealed that the placental metabolome did not differ when comparing the ASD and TD groups (*P* = 0.9393 under 9999 permutations) and ASD and Non-TD groups (*P* = 0.0529 under 9999 permutations) but did differ when comparing the Non-TD group to the TD group (*P* = 0.0231 under 9999 permutations). We then investigated the associations between individual placental metabolites and neurodevelopmental outcomes (Supplemental Table 2). However, no individual metabolites were associated with neurodevelopmental outcome at the FDR < 0.1 threshold.

We also investigated the multivariate associations between neurodevelopmental outcomes and the umbilical cord serum metabolome using PERMANOVA. We tested for and did not find differences in beta-dispersion by neurodevelopmental outcome (*P* = 0.70). In the covariate-adjusted model, neurodevelopmental outcome was not significantly associated with the cord serum metabolome (*R*^*2*^ = 0.020, *P* = 0.1221 under 9999 permutations).

### Exploratory mediation analysis

**FIGURE 1:**
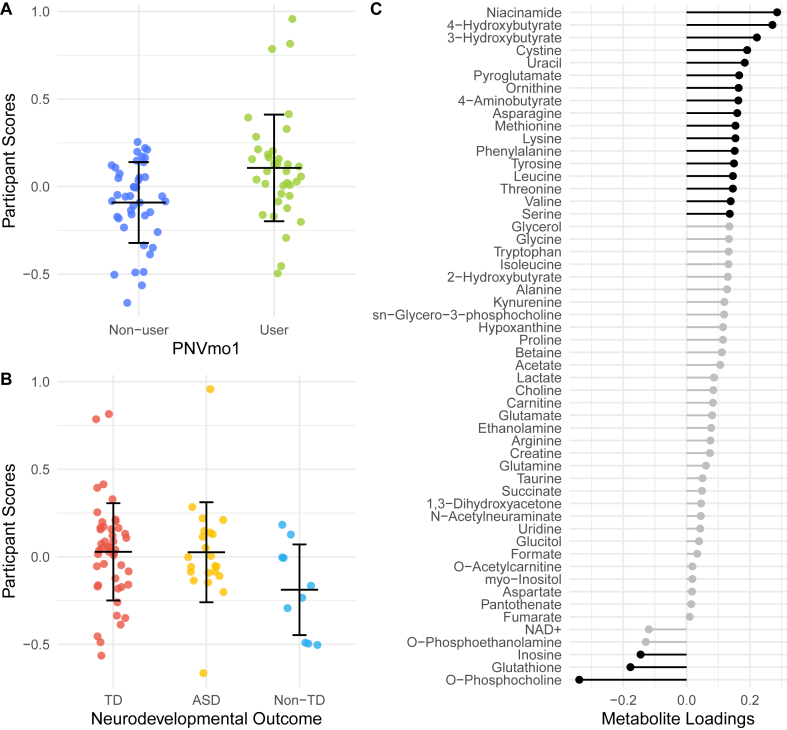
Redundancy analysis. Redundancy analysis was used to reduce the dimensionality of the placental metabolome into a single score associated with prenatal vitamin use in the first month of pregnancy (PNVmo1). Scores for individual placental samples are presented by (A) PNVmo1 and (B) neurodevelopmental outcome. Bars represent mean and SD. (C) Metabolite loadings for PC1 are presented and important metabolites are highlighted in black. Autism spectrum disorder (ASD), nontypical development (non-TD), and typically developing (TD).

Because the placental metabolome was associated with both PNVmo1 and neurodevelopmental outcome in this subset of MARBLES, we sought to determine if the placental metabolome could mediate the relationship between PNVmo1 and neurodevelopmental outcomes. We used RDA to reduce dimensionality and extract a placental metabolome score associated with PNVmo1 (RDA1, Figure 1A). After conditioning on covariates, PNVmo1 described 3.4% variation in the data (*P* = 0.0107 under 9999 permutations). The resulting RDA1 participant scores were lower in the Non-TD group compared with the TD group (Figure 1B) and this was significant after adjustment for covariates, and PNVmo1 (*P* = 0.03). No difference in score was observed between the ASD and TD groups (*P* = 0.83). Important metabolites whose squared loading was >$\frac{1}{54}$ are highlighted in black (Figure 1C). Metabolites that may be important contributors to the multivariate association between PNVmo1 and the placental metabolome identified by PERMANOVA and RDA included asparagine, cystine, leucine, lysine, methionine, *O*-phosphocholine, ornithine, phenylalanine, pyroglutamate, serine, threonine, tyrosine, uracil, and valine.

We conducted a mediation analysis to test our hypothesis that the placental metabolome explains a relationship between PNVmo1 and Non-TD likelihood, comparing Non-TD outcomes with TD outcomes as a reference. We did not find evidence of mediation by the placental metabolome (modeled as RDA1) when comparing Non-TD and TD outcomes in models adjusted for fetal sex, maternal age, maternal education, maternal self-reported race and ethnicity, home ownership, birth year, and maternal metabolic conditions (average causal mediation effect = –0.092, 95% CI: –0.222, –0.01, *P* = 0.07; average direct effect = –0.01, 95% CI: –0.248, 0.22, *P* = 0.95).

## Discussion

In this study, we investigated the associations between PNVmo1 and the placental and umbilical cord serum metabolomes in samples collected at birth. We found that PNVmo1 was associated with a small but significant estimated effect on the placental and umbilical cord serum metabolomes at birth, and this estimated effect was stronger in the placental metabolome and was driven by elevated concentrations of amino acids and other metabolites in the PNVmo1-exposed group. Notably, we did not observe the same associations with individual metabolites in the umbilical cord serum metabolome, although we have previously demonstrated that tissue-specific metabolomic profiles related to lipid metabolism and oxidative stress are highly correlated between placenta and umbilical cord serum in this cohort [16]. The placental and cord serum metabolomes represent metabolism in different compartments, with different metabolites and concentrations. The umbilical cord serum metabolome reflects the fetal phenotype at delivery. On the other hand, the placental metabolome is a window into a highly specialized organ with vital but temporary roles in nutrient metabolism and transfer. As the first organ to form, the placenta might be particularly sensitive to the nutritional environment in this period [33]. This builds on a growing body of evidence showing that the placenta is sensitive to maternal nutritional status before and during pregnancy [[34], [35], [36]]. Taken together, these findings suggest that placental metabolism could be more sensitive to periconceptional nutrient supplementation than fetal metabolism at delivery.

In this study, we also observed a difference in the placental metabolome between the TD and Non-TD groups. Surprisingly, we did not find evidence that the placental or cord serum metabolomes were associated with ASD in this subset of MARBLES. One reason we did not find a relationship between the placental and cord serum metabolomes and ASD could be because mothers in this cohort were recruited based on having an existing child with ASD. ASD has been shown to be heritable, and there may be a relationship between genetic risk and the placental and fetal metabolomes that tends to dominate the metabolic patterns we observe in this high-familial likelihood cohort [4]. Nevertheless, with only 10 mother–child dyads among the Non-TD group and 22 dyads among the ASD group in the placental sample, interpretation of these results must be taken with caution.

### Associations between PNVmo1 and placental metabolism

Here, we report that PNVmo1 is associated with a multivariate difference in the placental metabolome. To interpret the metabolome-wide differences observed in the placenta, we consider the metabolites identified as important contributors. Many of the amino acids and amino acid derivatives identified in this analysis, including asparagine, leucine, lysine, methionine, ornithine, phenylalanine, pyroglutamate, serine, threonine, tyrosine, and valine, were more abundant in placentas from the PNVmo1 exposed group. These included essential, conditionally essential, and nonessential amino acids. Elevated abundance of amino acids—especially essential isoleucine, leucine, lysine, methionine, phenylalanine, threonine, and valine (Table 2, FDR < 0.01)—suggests elevated placental amino acid transport, which regulated in part by micronutrient-sensing. In the placenta, mechanistic target of rapamycin (mTOR) is a key nutrient-sensor that regulates cell growth and proliferation, including amino acid transport [37]. Vitamin D and folic acid are among the nutrients sensed by mTOR. Through folate sensing by mTOR, maternal folate deficiency has been shown to result in reduced placental amino acid transport and restricted fetal growth [38,39]. Folate deficiency inhibits mTOR Complex 1 (mTORC1) and mTORC2, leading reduced expression of 2 important placental amino acid transporters [38]. Vitamin D, present in many prenatal vitamins, also regulates amino acid transport across the placenta through mTOR [40]. It is biologically plausible that early pregnancy nutrient status could influence placental biology even at delivery because nutrient status in early pregnancy might impact placental development. For instance, folic acid also plays a role in stimulating trophoblast invasion and angiogenesis in early pregnancy placental explants [41]. Additionally, changes in placental gene expression related to energy metabolism and intracellular transport were associated with maternal vitamin D levels measured at mid-pregnancy (but not at delivery) in a large birth cohort [42]. Thus, early availability of nutrients like folic acid and vitamin D in prenatal vitamins might program changes in placental function, including amino acid transport, leading to the differences observed here.

Amino acids including cystine, methionine, and serine also participate in 1-carbon metabolism and the transsulfuration pathway, which link B vitamin metabolism, methylation, amino acid metabolism, and glutathione synthesis [43]. Although glutathione concentrations were not significantly lower in the PNVmo1 group (Table 2), it was an important metabolite negatively associated with PNVmo1 (Figure 1C). At the same time, concentrations of placental pyroglutamate, an indicator of glutathione depletion [44], were elevated with PNVmo1. Although prenatal supplementation reduces the risk of dietary inadequacy, it is also associated with excessive intakes for some micronutrients, such as iron [6]. Excessive iron intake can induce oxidative stress. Indeed, markers of oxidative stress were shown to be higher in placentas from pregnant women who used iron supplements compared with those who did not use iron supplements in another study [45]. Thus, future research should investigate the role of PNV use on aspects of placental biology, including oxidative stress, especially in those who use PNV that exceed the recommended iron intake. This underscores the need for future research to identify optimal formulations for prenatal vitamin and mineral supplements.

We identified and quantified only a handful of micronutrients and their derivatives within the placental metabolome, including choline, *O*-phosphocholine, *sn*-glycero-3-phosphocholine, pantothenate, niacinamide, and NAD^+^. Notably, *O*-phosphocholine was strongly negatively loaded with PNVmo1 (Figure 1C) and significantly lower in placentas exposed to PNVmo1 (Table 2). *O*-phosphocholine has been found to be elevated with fetal growth restriction and preeclampsia [46,47]. Overall, we observe a few differences in micronutrients and their derivatives here. We speculate that this is because PNVmo1 exposure is associated with the placental metabolome through a variety of changes in placental biology, such as altered placental growth and vascularization, rather than through placental accumulation of micronutrients.

### Limitations and future directions

Although this secondary analysis was conducted in a well-characterized, prospective observational cohort [17], it has several limitations. First, the dosage and formulations of prenatal vitamins vary widely [48], adding variability that we were unable to account for to these results. Although most prenatal vitamins contain folic acid and iron, many formulations lack iodine, calcium, vitamin A, or choline, among other essential nutrients [49]. But even among micronutrients commonly found in prenatal supplements, the chemical form of some nutrients varies among supplements [48]. In this analysis, we were unable to investigate the impacts of individual prenatal vitamin formulations, which is a promising direction for future research. Among those using prenatal supplements, there exists a risk for both inadequate and excessive intake of certain micronutrients when accounting for total intake from food and supplements, including folic acid, iron, and zinc [6,7]. Thus, among the participants who used prenatal vitamins, there might be a spectrum of micronutrient adequacy, which could also contribute variability and hence uncertainty to these results. Second, this study was conducted in a cohort with enriched familial likelihood for ASD, so these findings might not be applicable in the broader population. Third, in this subset of a high-familial likelihood cohort, the sample size was small for analyses related to neurodevelopmental outcomes, particularly in the Non-TD group, comprising 10 dyads for the placental metabolome and 17 for the cord serum metabolome. Fourth, although a variety of potential confounding factors were assessed in this study, residual confounding by other unmeasured factors—like environmental toxicants or diet—cannot be ruled out. Fifth, it is important to consider that the placenta is a transient organ, and these placental samples were collected at birth. The metabolic state of the placenta at birth might not be reflective of placental metabolism during gestation.

Further research is necessary to confirm these findings in both high-familial likelihood and broader populations, as well as to identify the nutrient(s) and biochemical processes responsible for these changes in placental metabolism. For instance, in this small data set, PNVmo1 is likely associated with folic acid intake [5]. Further, ∼73% of pregnant women in the United States report using PNV that contain folic acid [50]. Thus, elevated free amino acid concentrations in the placenta associated with PNVmo1 could be related to increased folate availability in mothers using PNV generally containing folic acid. Likewise, nearly as many pregnant women in the United States report using PNV that contain iron [50], which could contribute elevated signs of oxidative stress observed here. Future studies that have much larger sample sizes; address the variability in prenatal vitamin composition, dosage, and timing; and are designed to specifically tease out these connections are needed.

In conclusion, this is the first study, to our knowledge, to investigate the effects of prenatal supplement use in the periconceptional period on the placental or umbilical cord serum metabolomes. We observed that PNVmo1 was associated with the placental metabolome—especially with amino acids—and that the placental metabolome was also associated with Non-TD outcomes but not with ASD outcomes. We also found that the umbilical cord serum metabolome was associated with PNVmo1. Whereas confirmation of these results in larger studies is needed, these findings could add nuance to our understanding of the role of PNV in developmental origins of health and disease and support a call-to-action to study and eventually regulate PNV formulations.

## Author contributions

The authors’ contributions were as follows – RJS, SO, IH-P, CKW, CMS: designed research; MP, MM, CKW: conducted research; MP: analyzed data; DJT: provided critical statistical insights; MP, CMS: wrote the article; CMS: primary responsibility for final content; and all authors: read and approved the final manuscript.

## Data availability

Data described in the manuscript, code book, and analytic code will be made available upon request pending application and approval to protect participant privacy.

## Funding

The project described was supported by grant numbers T32ES007059, R21ES028129, R01ES020392, U24ES028533, R24ES028533, R01ES028089, R01ES025574, and P01ES011269 from NIH NIEHS, as well as the University of California Davis MIND Institute Intellectual and Developmental Disabilities Research Center (IDDRC) U54-HD079125 from the NIH NICHD. Its contents are solely the responsibility of the authors and do not necessarily represent the official views of the NIEHS, NICHD, or NIH. This work was also supported by US EPA STAR grants RD#829388 and RD#833292, the USDA National Institute of Food and Agriculture Hatch project 1021411, and the Simons Foundation Autism Research Initiative 863967. CMS would also like to acknowledge the Kinsella Endowed Chair in Food, Nutrition, and Health.

## Conflict of interest

The authors report no conflicts of interest.
