# Supplementary material for: Placental Metabolism Is Linked to Prenatal Vitamin Supplement Use in the First Month of Pregnancy in the MARBLES Cohort
Source: J Nutr. 2025 May 23;155(7):2185–94. doi: 10.1016/j.tjnut.2025.05.016 (PMC12673020; doi:10.1016/j.tjnut.2025.05.016)
Supplement: Multimedia component 1 [file mmc1.pdf]

SUPPLEMENTARY INFORMATION

Placental metabolism is linked to prenatal vitamin supplement use in the first month of pregnancy in the MARBLES cohort

Mariana Parenti et al.

CONTENTS

Supplementary Figure 1: Directed Acyclic Graph

Supplementary Table 1. Associations between prenatal vitamin use in the first month of pregnancy (PNVmo1) and umbilical cord serum metabolite concentrations (µmol/L)

Supplementary Table 2. Associations between neurodevelopmental outcomes and placental metabolite concentrations (nmol/g).

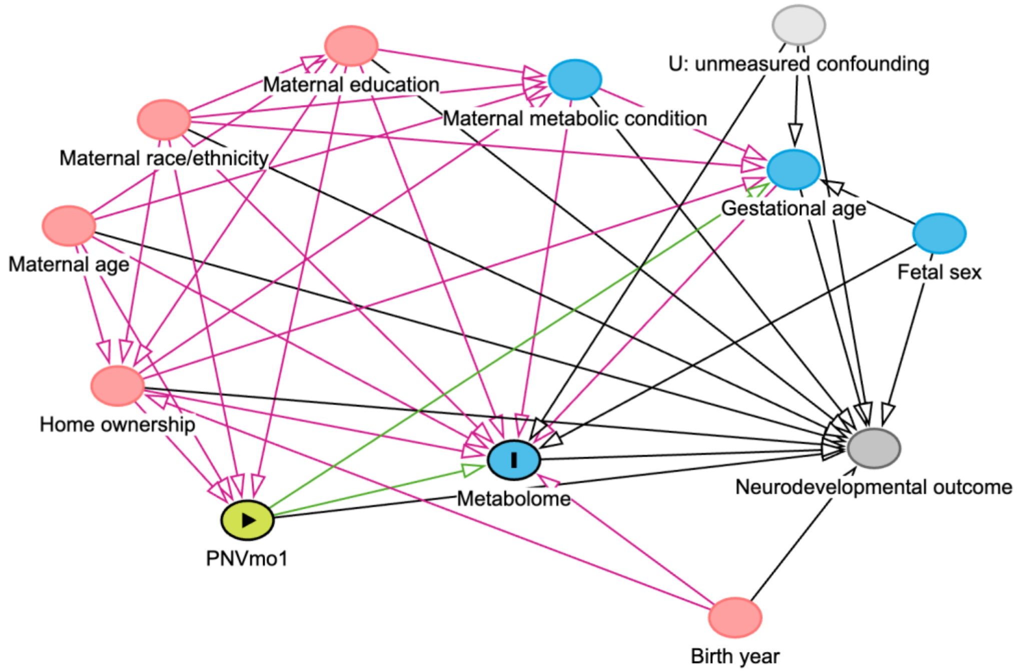

Supplementary Figure 1. The directed acyclic graph (DAG) for the associations between prenatal vitamin use in the first month of pregnancy (PNVmo1), the metabolome, and neurodevelopmental outcome. Unmeasured latent variables are designated with "U". In the DAG, maternal race/ethnicity serves as a proxy for maternal experience of structural inequity. Likewise, home ownership is a proxy for socioeconomic status.

Supplementary Table 1. Associations between prenatal vitamin use in the first month of pregnancy (PNVmo1) and umbilical cord serum metabolite concentrations (µmol/L)

Metabolite	No PNVmo1[a] (*N*=67)	PNVmo1 [a] (*N*=65)	R^2 [b]	*p-value*	FDR
2-Aminobutyrate	22.1 (17.3, 27.4)	24.0 (19.5, 29.3)	0.0022	0.6126	0.8695
2-Hydroxybutyrate	42.4 (27.6, 53.3)	47.5 (36.7, 55.3)	0.0070	0.3356	0.6868
2-Oxoisocaproate	6.9 (5.8, 8.5)	7.5 (6.6, 9.4)	0.0151	0.1034	0.5055
3-Hydroxybutyrate	122.1 (54.4, 225.3)	141.3 (70.6, 246.4)	0.0037	0.4982	0.8314
3-Hydroxyisobutyrate	19.0 (14.5, 21.4)	19.2 (17.1, 21.5)	0.0085	0.2846	0.6868
3-Methyl-2-oxo-butanoic acid	15.2 (12.8, 18.0)	16.8 (13.5, 20.5)	0.0325	0.0168	0.3212
3-Methyl-2-oxovalerate	7.9 (6.8, 10.1)	8.5 (6.8, 10.2)	<0.0001	0.9782	0.9782
Acetate	36.4 (32.7, 45.1)	35.6 (31.8, 41.6)	0.0073	0.3258	0.6868
Acetone	4.3 (2.8, 5.0)	5.0 (4.2, 5.4)	0.0337	0.0301	0.3212
Alanine	478.2 (399.1, 529.3)	461.7 (397.2, 512.9)	0.0053	0.3746	0.6868
Arginine	86.4 (69.1, 100.5)	89.8 (72.9, 105.9)	0.0097	0.2691	0.6868
Ascorbate	25.5 (16.7, 32.8)	28.1 (19.8, 36.6)	0.0003	0.8310	0.9671
Asparagine	56.1 (49.2, 63.2)	57.7 (49.1, 64.3)	0.0058	0.3501	0.6868
Aspartate	30.0 (22.7, 36.9)	29.2 (21.7, 36.1)	0.0135	0.1920	0.6868
Betaine	29.3 (24.6, 33.1)	30.3 (26.3, 34.4)	0.0001	0.9231	0.9671
Choline	35.8 (29.4, 42.4)	36.0 (31.6, 43.1)	0.0068	0.3605	0.6868
Creatine	50.5 (42.0, 63.5)	48.6 (42, 60.7)	0.0218	0.0971	0.5055
Creatinine	41.6 (37.5, 50.2)	43.5 (39.4, 49.5)	0.0007	0.7715	0.9671
Cystine	30.2 (25.4, 38.4)	32.5 (27.1, 40.7)	0.0002	0.8860	0.9671
Dimethyl sulfone	3.7 (2.3, 5.4)	3.7 (2.6, 5.2)	0.0003	0.8426	0.9671
Ethanolamine	35.2 (31.0, 40.5)	35.4 (30.6, 41.0)	0.0123	0.2099	0.6868
Formate	20.9 (17.9, 24.4)	21.0 (18.6, 24.0)	0.0007	0.7692	0.9671
Glucose	3083.3 (2642.9, 3583.2)	3220.2 (2778.4, 3718.4)	0.0004	0.8200	0.9671
Glutamate	105.2 (71.4, 147.4)	106.3 (76.8, 150.4)	0.0069	0.3408	0.6868
Glutamine	545.9 (510.7, 594.1)	549.0 (503.6, 612.6)	0.0001	0.9178	0.9671
Glycine	283.9 (258.0, 319.8)	280.7 (252.2, 303.2)	0.0328	0.0381	0.3212
Histidine	87.1 (77.4, 94.8)	83.6 (78.9, 90.7)	0.0041	0.4597	0.8091
Hypoxanthine	15.1 (10.9, 29.6)	13.0 (9.6, 23.5)	0.0133	0.1792	0.6868
Isoleucine	73.6 (66.3, 80.4)	75.0 (68.1, 81.5)	0.0003	0.8505	0.9671
Lactate	4635.0 (3621.6, 5968.1)	4437.8 (3809, 6241.1)	0.0028	0.5110	0.8314
Leucine	115.6 (101.2, 124.4)	115.8 (105.2, 126.1)	<0.0001	0.9652	0.9782
Lysine	338.5 (307.1, 376.4)	332.6 (306.3, 368.9)	0.0001	0.8873	0.9671
Methionine	35.2 (32.0, 39.5)	34.8 (30.9, 38.2)	0.0082	0.2722	0.6868
myo-Inositol	64.8 (53.3, 71.8)	57.3 (53.0, 71.1)	0.0298	0.0438	0.3212
Ornithine	110.0 (94.5, 129.5)	111.0 (95.4, 135.2)	0.0020	0.6055	0.8695
Phenylalanine	73.4 (66.4, 81.1)	72.2 (65.1, 78.5)	0.0088	0.2802	0.6868
Proline	151.8 (133.7, 167.5)	146.7 (134.5, 164.6)	0.0234	0.0710	0.4463
Pyroglutamate	38.4 (30.0, 48.4)	37.3 (30.0, 45.4)	0.0320	0.0294	0.3212

Metabolite	No PNVmo1[a] (*N*=67)	PNVmo1 [a] (*N*=65)	R^2 [b]	*p-value*	FDR
Pyruvate	26.3 (14.9, 41.8)	41.7 (18.5, 67.6)	0.0485	0.0047	0.2068
Serine	169.4 (153.0, 193.6)	171.2 (162.3, 197.3)	0.0008	0.7353	0.9671
Taurine	225.1 (174.0, 268.1)	213.3 (170.8, 262.2)	0.0127	0.1986	0.6868
Threonine	209.2 (180.6, 238.5)	223.8 (193.3, 249.6)	0.0028	0.5291	0.8314
Tyrosine	72.9 (61.2, 79.2)	70.2 (62.2, 80.0)	0.0058	0.3604	0.6868
Valine	250.8 (222.8, 276.2)	261.0 (227.6, 276.6)	0.0027	0.5567	0.8446

[a] Median and interquartile range.

[b] PERMANOVA was conducted on log-transformed metabolite concentrations under 9999 permutations. All models were adjusted for birth year, maternal age, maternal self-reported race and ethnicity, home ownership, maternal education, maternal metabolic condition, and fetal sex.

Abbreviations: PNVmo1: prenatal vitamin/mineral supplement use in the first month of pregnancy; No PNVmo1: no prenatal vitamin/mineral supplement use in the first month of pregnancy; FDR, False Discovery Rate; R^2: partial coefficient of determination.

Supplementary Table 2. Associations between neurodevelopmental outcomes and placental metabolite concentrations (nmol/g).

**Metabolite**	**TD [a] (*N*=42)**	**ASD [a] (*N*=22)**	**Non-TD [a] (*N*=10)**	R^{2} [b]	***p-value***	**FDR**
1,3-Dihydroxyacetone	16.8 (8.2, 30.2)	17.0 (7.3, 34.8)	12.9 (0.6, 22.7)	0.0909	0.0273	0.2992
2-Hydroxybutyrate	30.4 (18.9, 35.7)	30.1 (23.3, 34.2)	31.7 (22.0, 38.9)	0.0166	0.4696	0.7727
3-Hydroxybutyrate	155.2 (69.4, 297.6)	135.8 (89.1, 204.2)	238.4 (130.0, 374.0)	0.0191	0.4904	0.7727
4-Aminobutyrate	25.7 (18.5, 33.0)	25.2 (19.4, 34.6)	29.3 (21.9, 34.5)	0.0060	0.8100	0.9405
4-Hydroxybutyrate	40.1 (25.9, 56.1)	36.2 (26.8, 49.1)	3.5 (0, 45.5)	0.1309	0.0072	0.1944
Acetate	273.9 (211.8, 331.9)	302.3 (239.2, 364.5)	213.3 (154.8, 267.0)	0.0039	0.8360	0.9405
Alanine	929.5 (785.7, 1026.8)	918.3 (844.1, 1017.3)	851.7 (811.4, 999.8)	0.0244	0.3487	0.7532
Arginine	184.9 (149.4, 241.)	173.1 (149.7, 207.8)	171.4 (124.7, 195.4)	0.0429	0.1653	0.5886
Asparagine	163.2 (137.8, 201.1)	162.8 (131.6, 186.7)	156.5 (132.8, 177.0)	0.0287	0.2991	0.6847
Aspartate	1015.8 (850.3, 1187.1)	1026.0 (861.5, 1183.0)	861.3 (717.4, 977.5)	0.0953	0.0255	0.2992
Betaine	29.8 (22.6, 35.5)	29.8 (20.2, 34.2)	28.2 (24.3, 31.6)	0.0079	0.7269	0.9046
Carnitine	131.5 (111.3, 151.4)	138.4 (99.6, 161.7)	131.7 (102.0, 157.1)	0.0152	0.5681	0.7727
Choline	1663.9 (1465.9, 2011.9)	1603.5 (1238, 1757.4)	1644.7 (1560.2, 1960.1)	0.0035	0.8802	0.9416
Creatine	385.7 (312.6, 484.5)	463.6 (387.6, 544.8)	406.6 (385.0, 523.3)	0.0603	0.1051	0.5886
Cystine	35.4 (27.5, 47.9)	28.4 (22.9, 45.5)	40.4 (21.0, 45.7)	0.0196	0.4630	0.7727
Ethanolamine	1003.6 (890.1, 1162.5)	978.8 (758.4, 1220.4)	764.4 (676.5, 1251.4)	0.0136	0.5867	0.7727
Formate	198.1 (177.3, 228.8)	203.7 (173.1, 244.5)	176.7 (157.0, 203.7)	0.0031	0.8893	0.9416
Fumarate	36.3 (27.8, 52.9)	43.5 (28.6, 54.4)	34.7 (28.9, 46.3)	0.0009	0.9706	0.9904
Glucitol	925.7 (758.6, 1119.6)	1054.2 (782.5, 1305.1)	801.5 (584.9, 1048.2)	0.0497	0.1564	0.5886
Glutamate	2626.7 (2443.0, 3070.8)	2769.1 (2299.5, 2944)	2558.1 (2318.8, 3089.6)	0.0099	0.6776	0.8712
Glutamine	915.1 (836.1, 1004.3)	854.1 (769.4, 937.9)	859.4 (741.5, 874.2)	0.0295	0.2887	0.6847
Glutathione	96.5 (63.8, 121.8)	106.7 (53.7, 169.7)	115.8 (106.9, 131.6)	0.0076	0.7371	0.9046
Glycerol	1865.0 (1535.6, 2387.2)	1718.1 (1419.3, 2027.8)	1938.8 (1433.5, 2253.8)	0.0055	0.8058	0.9405
Glycine	1144.5 (1004.8, 1366.9)	1184.5 (950.9, 1308.9)	1038.7 (921.4, 1328.3)	0.0159	0.5247	0.7727
Hypoxanthine	655.2 (523.7, 787.4)	595.6 (492.7, 708.1)	557.3 (498.8, 640.0)	0.0145	0.5679	0.7727
Inosine	199.8 (138.9, 281.5)	253.9 (198.7, 300.4)	229.3 (207.4, 332.6)	0.0394	0.1828	0.5886
Isoleucine	169.9 (144.0, 190.0)	170.4 (146.3, 182.5)	158.3 (141.6, 182.6)	0.0323	0.2496	0.6847
Kynurenine	31.9 (16.6, 42.6)	26.6 (16.0, 37.6)	23.4 (15.5, 38.1)	0.0035	0.8804	0.9416

Lactate	19028.3 (16672.5, 21498.3)	19070.1 (18086.4, 23077.2)	18985.8 (17617.9, 20689.4)	0.0150	0.5381	0.7727
Leucine	301.3 (258.1, 344.3)	297.7 (246.1, 330.8)	260.1 (249.2, 320.2)	0.0437	0.1342	0.5886
Lysine	309.1 (262.7, 340.2)	296.6 (233.7, 333.4)	235.7 (205.0, 284.9)	0.0790	0.0277	0.2992
Methionine	87.8 (75.4, 105.9)	86.0 (70.8, 101.4)	85.1 (71.7, 90.7)	0.0357	0.1853	0.5886
myo-Inositol	1608.1 (1324.4, 1760.4)	1424.0 (1278.1, 1774.0)	1455.4 (1219.1, 1644.5)	0.0319	0.2923	0.6847
N-Acetylneuraminate	262.3 (212.5, 368.7)	285.1 (210.6, 317.0)	232.6 (174.4, 306.8)	0.0216	0.4520	0.7727
NAD^+	59.8 (47.6, 80.6)	61.5 (51.1, 68.7)	61.0 (44.6, 96.4)	0.0101	0.5587	0.7727
Niacinamide	77.1 (58.8, 92.5)	71.9 (55, 92.8)	59.1 (1.7, 76.0)	0.0799	0.0525	0.4725
O-Acetylcarnitine	78.1 (61.3, 94.4)	65.6 (62.1, 103.0)	78.6 (59.2, 94.1)	0.0141	0.5775	0.7727
O-Phosphocholine	140.2 (106.1, 176.5)	125.8 (92.5, 159.1)	125.9 (111.4, 187.1)	0.0182	0.4513	0.7727
O-Phosphoethanolamine	520.8 (258.1, 659.1)	477.2 (352.4, 691.8)	389.5 (316.8, 573.8)	0.0001	0.9975	0.9975
Ornithine	51.6 (42.6, 59.5)	49.4 (39.0, 61.6)	50.1 (42.0, 64.9)	0.0006	0.9721	0.9904
Pantothenate	18.6 (13.8, 25.7)	14.9 (12.7, 22.1)	12.4 (9.2, 16.1)	0.1151	0.0060	0.1944
Phenylalanine	177.3 (148.4, 193.7)	162.3 (151.8, 185.1)	157.9 (139.8, 193.3)	0.0289	0.2650	0.6847
Proline	358.7 (308.3, 436.3)	380.6 (333.8, 429.9)	351.3 (343.9, 384.3)	0.0357	0.2239	0.6717
Pyroglutamate	47.2 (35.5, 58.9)	48.1 (39.0, 67.4)	57.0 (41.4, 63.9)	0.0411	0.1644	0.5886
Serine	458.1 (373.1, 539.1)	427.0 (368.2, 520.2)	426.8 (349.2, 483.0)	0.0265	0.3043	0.6847
sn-Glycero-3-phosphocholine	155.3 (105.5, 246.5)	157.5 (69.6, 222.8)	156.4 (88.7, 353.9)	0.0164	0.4882	0.7727
Succinate	217.4 (158.5, 251.5)	211.4 (182.2, 294.2)	264.5 (192.8, 332.5)	0.0135	0.5613	0.7727
Taurine	3121.2 (2760.4, 3701.4)	3040.6 (2447.9, 3354.2)	3089.9 (2408.3, 3686.9)	0.0051	0.8223	0.9405
Threonine	450.0 (384.7, 554.4)	429.1 (391.8, 495.5)	405.1 (348.9, 454.4)	0.0439	0.1693	0.5886
Tryptophan	20.1 (12.6, 26.4)	28.0 (18.0, 32.5)	25.0 (21.4, 28.2)	0.0417	0.1835	0.5886
Tyrosine	195.1 (166.1, 225.0)	188.6 (168.2, 210.5)	189.4 (157.8, 224.8)	0.0170	0.4848	0.7727
Uracil	368.9 (256.2, 480.6)	306.7 (228.6, 380.5)	252.2 (227.5, 416.6)	0.0210	0.4617	0.7727
Uridine	127.8 (100.8, 148.3)	112.0 (83.1, 144.7)	121.0 (84.2, 161.7)	0.0686	0.0707	0.5454
Valine	399.4 (344, 440.1)	387.5 (341.4, 426.4)	359.1 (313.9, 414.5)	0.0414	0.1559	0.5886

[a] Median and interquartile range.

[b] PERMANOVA was conducted on log-transformed metabolite concentrations under 9999 permutations. All models were adjusted for birth year, maternal age, maternal self-reported race and ethnicity, home ownership, maternal education, fetal sex, maternal metabolic condition, and prenatal vitamin/mineral supplement use in the first month of pregnancy.

Abbreviations: Typical Development (TD); Autism Spectrum Disorder (ASD); Non-Typical Development (Non-TD); FDR, False Discovery Rate; R^2: partial coefficient of determination.
